# Plant community responses to environmentally friendly piste management in northeast Iran

**DOI:** 10.1002/ece3.5388

**Published:** 2019-06-19

**Authors:** Mohammad Bagher Erfanian, Hamid Ejtehadi, Jamil Vaezi, Hamid Moazzeni, Farshid Memariani, Mohammad Firouz‐Jahantigh

**Affiliations:** ^1^ Department of Biology, Quantitative Plant Ecology and Biodiversity Research Lab Faculty of Science Ferdowsi University of Mashhad Mashhad Iran; ^2^ Department of Botany, Research Center for Plant Sciences Ferdowsi University of Mashhad Mashhad Iran

**Keywords:** biodiversity, degraded alpine lands, overgrazing, piste, restoration

## Abstract

It is well‐known that pistes have adverse effects on alpine ecosystems. Previous studies urged that pistes should be installed and managed in the ways to minimize negative impacts on natural habitats. However, the impacts of this type of management on the plant communities are not widely studied. The aim of this study was to examine species composition and biodiversity changes in an environmentally friendly managed piste in northeast Iran. This piste has been established in a previously degraded alpine landscape. For the vegetation survey, we sampled 44 within and 28 off‐piste plots. Except for the piste management, other environmental factors were similar between the piste and off‐piste plots. Dominant species were determined, and variation in community composition of the two areas was visualized. Also, native species, phylogenetic, and functional Hill diversity of the two areas were compared. The results showed that there was a moderate differentiation in the species composition of the piste and off‐piste. Two palatable species (i.e., *Bupleurum falcatum* and *Melica persica*) were dominant in the piste and were not recorded in the off‐piste. The diversity calculations results showed that the species diversity of the piste was higher than that of the off‐piste. Phylogenetic diversity at the level of frequent and dominant plants showed a similar result. The piste had a higher functional diversity in terms of functional richness, and functional diversity of frequent and dominant plants. Our findings imply, after 10 years, species, phylogenetic, and functional diversity of the piste is significantly improved. Environmentally friendly piste management (EFPM) induced species composition change that led to emerging species that were absent in the off‐piste. We can conclude that EFPM led to restoration of a degraded landscape. Long‐term impacts of EFPM are still unknown, therefore, caution should be undertaken regarding the installation of new environmentally friendly pistes in other areas.

## 1 INTRODUCTION

Land‐use changes and grazing are the main drivers for habitat degradation in the mountainous ecosystems (Bhatta, Grytnes, & Vetaas, 2018; Lembrechts et al., 2017; Li, Fassnacht, Storch, & Bürgi, 2017; Lu et al., 2017; Pauchard et al., 2009). Furthermore, climate change and invasion of lower‐elevation plants threaten established plant communities of these areas (Walther et al., 2002). Piste management is among the commonly investigated types of land‐use changes considered to be an alpine habitat disturbing event (Roux‐Fouillet, Wipf, & Rixen, 2011).

There is a considerable amount of reports on the negative impacts of pistes. The outcomes of piste management are damaged soil, delayed flowering stage, altered vegetation structure, reduced biodiversity, and decreased productivity (Kangas, Tolvanen, Kälkäjä, & Siikamäki, 2009; Steinbauer, Kreyling, Stöhr, & Audorff, 2018; Wipf, Rixen, Fischer, Schmid, & Stoeckli, 2005; WWF, 2005). Piste management not only affects the plant species but also endangers fauna of alpine habitats (Caprio, Chamberlain, Isaia, & Rolando, 2011; Rolando, Caprio, Rinaldi, & Ellena, 2006; Sato et al., 2014). Due to the heavy habitat destructing impacts of pistes, researchers have urged that pistes should be managed in the ways that minimizes their negative effects on natural ecosystems (George, 2003; Roux‐Fouillet et al., 2011; Wipf et al., 2005). However, the impacts of this advised management type on plant communities are not widely understood.

Iran is a mountainous country, where (over)grazing has prominently affected the alpine habitats (Noroozi, Akhani, & Breckle, 2008; Noroozi et al., 2018). Binalood Mountains are located in northeast Iran. The climate of these mountains is semiarid (Shabanian et al., 2012). Early and overgrazing have caused the degradation of natural vegetation in this mountain range (Memariani, Joharchi, Ejtehadi, & Emadzade, 2009). A piste has been established in these degraded lands since 2008. Except for the limited machine‐grading, most of the common degrading piste managements (e.g., artificial snow, vegetation sowing, mowing) have not been applied in this piste. We use term “environmentally‐friendly piste management,” abbreviated as “EFPM” hereafter, to distinguish this type of management from “nature‐friendly piste management” that was used by Kašák, Mazalová, Šipoš, and Kuras (2013). Neither machine‐grading nor species cutting was performed in the nature‐friendly piste management (Kašák et al., 2013). This piste provides a natural experiment for the assessment of the consequences of EFPM in the area. To the best knowledge of the authors, there is no previous study for assessing the impacts of pistes on restoration of such degraded lands.

The piste has been established in degraded alpine land since 2008, it can be supposed that different management of the piste and neighboring lands has different effects on plant communities in the area. We hypothesize that EFPM can mitigate habitat degradation. Piste management affects the plant species composition and biodiversity (Allegrezza, Cocco, Pesaresi, Courchesne, & Corti, 2017; Roux‐Fouillet et al., 2011; Wipf et al., 2005). Therefore, vascular plant species composition along with native biodiversity was considered in this study. We evaluated multiple facets of biodiversity including species, phylogenetic, and functional diversity. Functional diversity was measured using five traits that we predicted to vary between the piste and off‐piste. Moreover, phylogenetic diversity was considered because it is related to evolutionary history and is also a measure of conservation priority (Faith, 2016).

## 2 MATERIALS AND METHODS

### 2.1 Study area

The study area (~400 hectares) was located in northeast Iran—the alpine area of Shirbad Summit in Binalood Mountains. The elevation ranges between 2,700 and 3,300 m a. s. l. The central point of the study area is positioned in 36.310°N and 059.058°E. Annual mean temperature and annual precipitation of the area are 2.6°C and 361 mm, respectively, according to the WorldClim 2 data (Fick & Hijmans, 2017). The area has a Mediterranean climate condition which is characterized by relatively high winter‐precipitation and several summer dry months (Djamali et al., 2011). The study area consists of shale and dark‐gray phyllite rocks (Geological Survey of Iran, 1986), representing a poorly developed soil. The area is a pasture with a long history of overgrazing that has formed degraded‐unvegetated gaps in it.

We surveyed two adjacent north‐facing landscapes with different land uses. There is approximately 3 km distance between central points of the two. One of these was a degraded pasture (hereafter named off‐piste); the other was a newly established piste. Limited machine‐grading, late‐season grazing by sheep and goats, and selective cutting of the aerial parts of *Verbascum* sp. are the managements that have affected the vegetation in the piste in the past 10 years (2008–2018). Both areas had similar grazing regime (i.e., early‐ and overgrazing) before launching the piste. Also, the climate and soil composition of the two landscapes are similar.

### 2.2 Sampling

Sampling was conducted during 2017–2018 growing seasons. The 2,700–3100 m a. s. l. elevation range, the piste's elevation range, was selected for the survey. The vegetation in the study area was herbaceous, so 1 m^2^ quadrats were used for sampling. A total number of 44 and 28 random plots were recorded in the piste and off‐piste, respectively. In each plot, a list of vascular plant species and their canopy cover were recorded. Additionally, functional traits that are related to persistence, establishment, reproductive success, dispersal strategy, and grazing tolerance with 40 replicates were noted for the recorded plants. These traits were Raunkiaer's life‐form, plant height, seed dispersal—data for this trait are also obtained from the TRY database (Kattge et al., 2011), seed numbers, and spininess.

### 2.3 Data analysis

#### 2.3.1 Species composition

We calculated the relative importance value index (RIV; the mean of relative frequency and relative canopy cover) to determine dominant plants in the piste and off‐piste. Bar plots showing ten species with the highest RIV values were drawn using the ggplot2 package (Wickham, 2009). Furthermore, transformation‐based principal component analysis (tb‐PCA) was used to visualize the species composition variation among the piste and off‐piste plots. To remove the effects of double‐zeros in the analysis, data were transformed using the Hellinger transformation (Legendre & Legendre, 2012). The *decostand* function in the vegan package was used for this purpose. Then, PCA was performed on the transformed data by using the *rda* function in the vegan package.

#### 2.3.2 Biodiversity

Data obtained from the piste and off‐piste plots were pooled separately to calculate the biodiversity. To examine the restoring effects of piste, we considered only the native species. Hereafter, the term (bio)diversity indicates native diversity. We used Hill diversity indices for our calculations (Chao, Chiu, & Jost, 2014). These indices were chosen because they are considered the standard measures of diversity (Chao, Chiu, et al., 2014; Ellison, 2010). To compare Hill diversity in the two areas, we used the proposed approach of Chao and Jost (2012). This method is called coverage‐based approach. It uses rarefaction and extrapolation procedures to estimate the diversity of multiple assemblages at the same sampling completeness (Chao & Jost, 2012). Therefore, in this approach, the effect of unequal sample numbers is eliminated form diversity comparisons (Chao, Gotelli, et al., 2014). This method has been applied to Hill species diversity and phylogenetic Hill diversity indices (Chao et al., 2015; Chao, Gotelli, et al., 2014; Hsieh, Ma, & Chao, 2016). However, it is not adapted to functional Hill diversity.

We used the protocol of Chao, Gotelli, et al. (2014) to calculate the base coverage level. It was calculated using the *estimateD* function in the iNEXT package (Hsieh et al., 2016). Then, species and phylogenetic diversity calculations were estimated at this computed coverage level. Hill species diversity in the zero (*q* = 0), first (*q* = 1), and second (*q* = 2) orders (or species richness, the exponential of the Shannon–Wiener index, and the reciprocal of the Gini–Simpson indices, respectively) were computed using the *estimateD* function in the iNEXT package (Chao, Gotelli, et al., 2014; Hsieh et al., 2016) in R ver. 3.5 (R Core Team, 2018). By considering these diversity indices, we were able to compare the diversity at the level of rare, frequent, and dominant species (Chao, Chiu, et al., 2014). The 95% confidence intervals (CI) for these indices were computed by using the internal bootstrapping procedure of the *estimateD* function.

The phylogenetic tree of the recorded native plant species in the study area was obtained by using the method proposed by Qian and Jin (2016). Then, phylogenetic Hill diversity at the zero (*q* = 0), first (*q* = 1), and second (*q* = 2) orders with the 95% CI was calculated using the *estimatePD* function in the iNEXT‐PD package (Chao et al., 2015; Hsieh et al., 2016) in R.

The mean of the quantitative traits along with the qualitative ones was used in functional Hill diversity calculations. To do so, using all of the traits, the Gower distance (Legendre & Legendre, 2012) were computed using the *gowdis* function in the FD package (Laliberté, Legendre, & Shipley, 2014). This distance matrix and native species frequency data were used to calculate functional Hill diversity (i.e., *q* = 0, 1, and 2 orders). We used the available R code of Chiu and Chao (2014) to calculate these indices. The bootstrapped‐95% C.I. for each functional diversity index was computed using R. The graphs depicting the results of biodiversity calculations were drawn using the ggplot2 R package (Wickham, 2009).

## 3 RESULTS

### 3.1 Species composition

**Figure 1:**
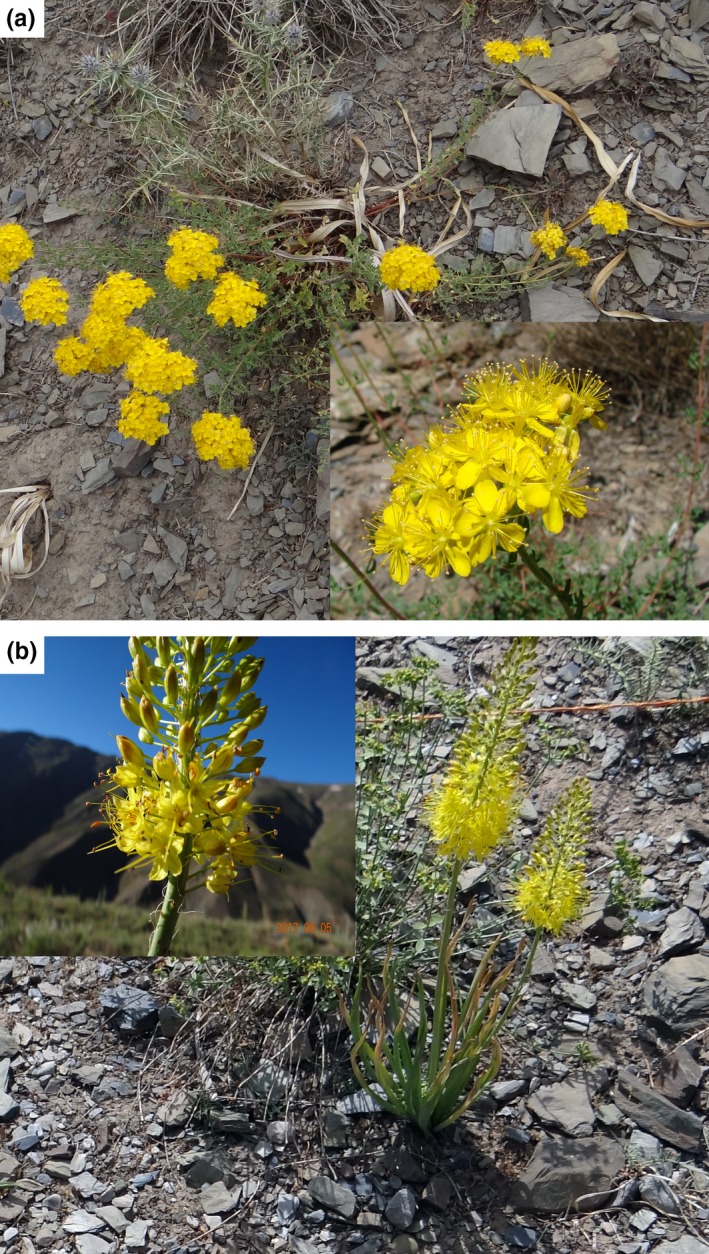
The dominant plant species in the study area. (a) *Hypericum scabrum*, the dominant species in the piste; (b) *Eremurus stenophyllus*, the dominant species in the off‐piste

**Figure 2:**
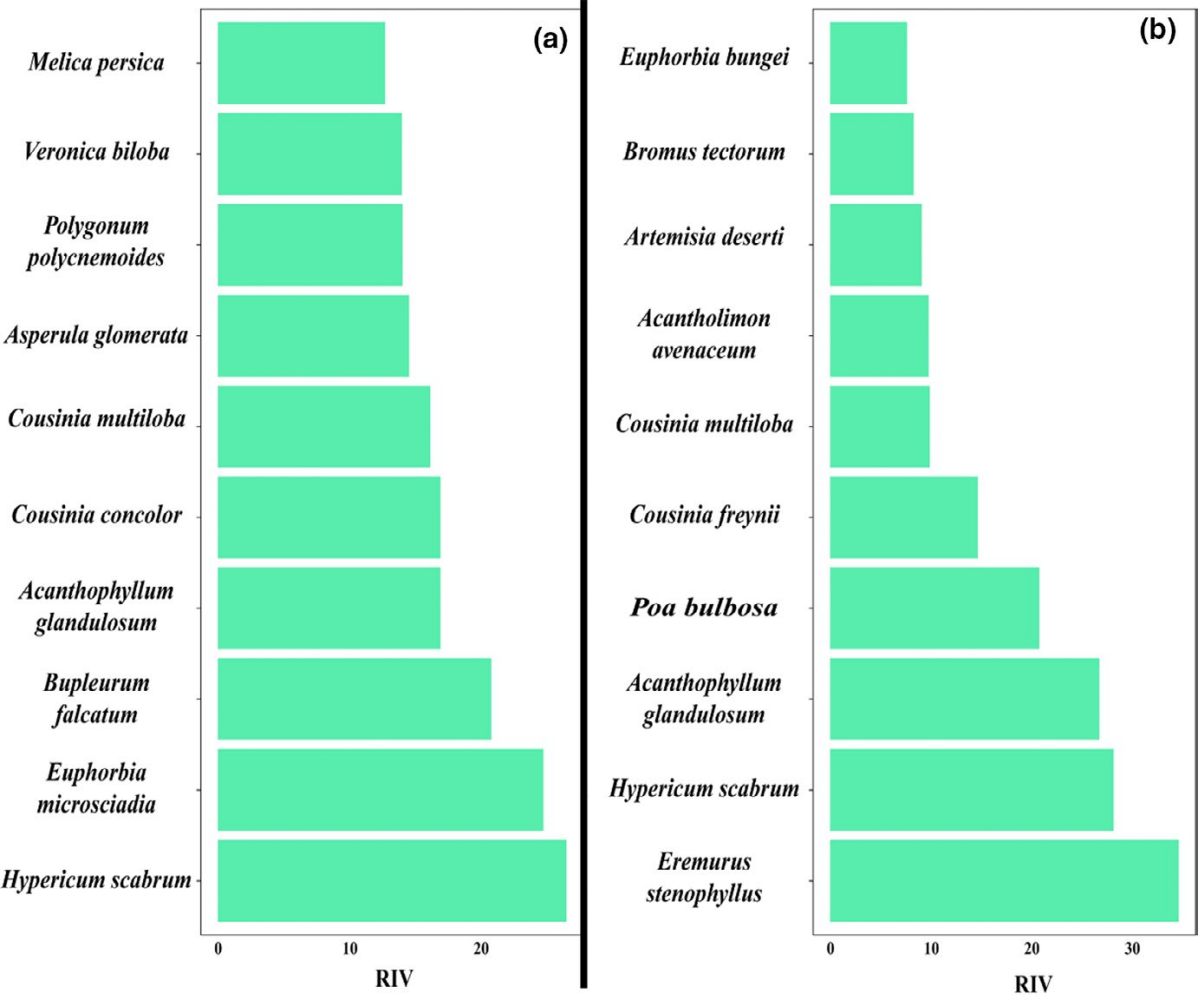
Relative importance value (RIV) of 10 dominant plant species in the piste (a) and off‐piste (b). All sample plots were pooled to calculate RIV

**Figure 3:**
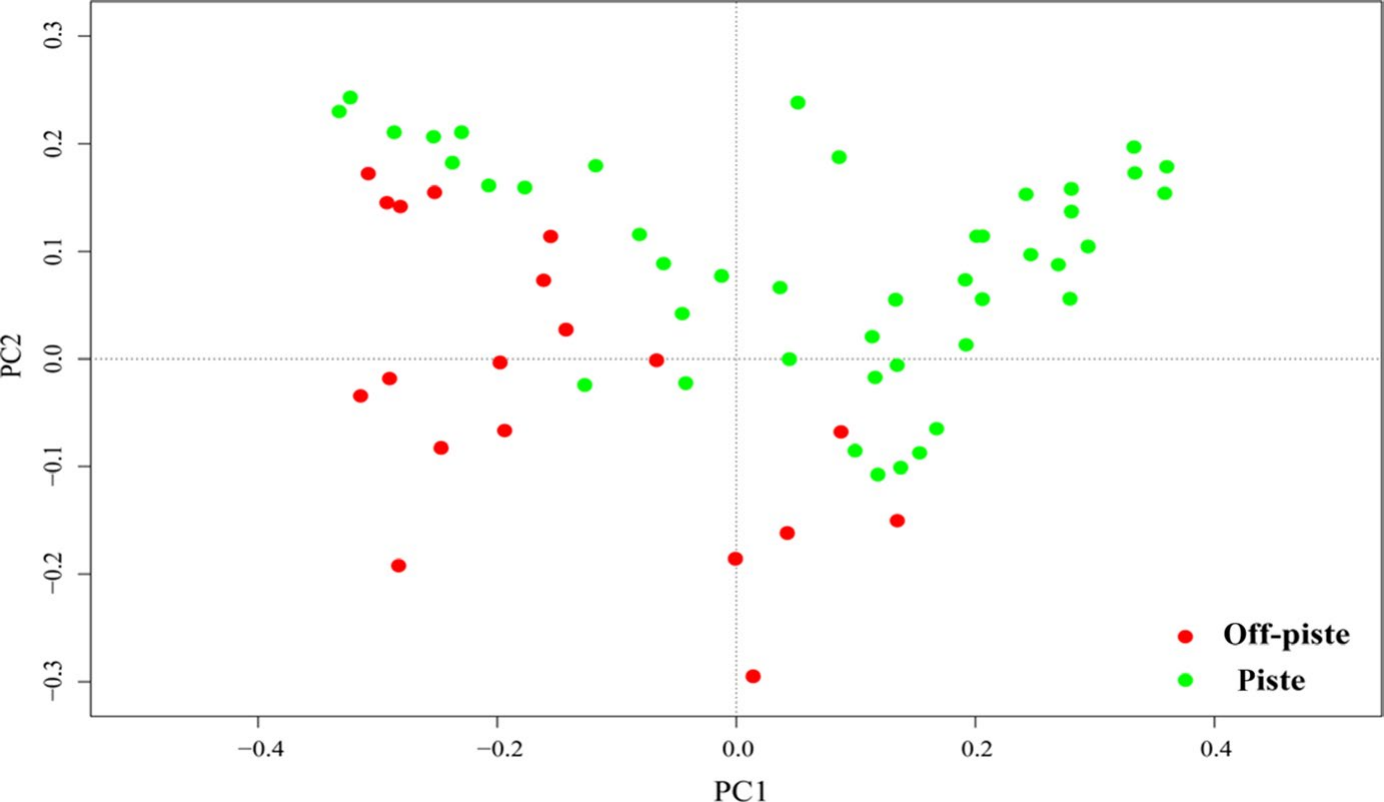
Transformation‐based principal component analysis (tb‐PCA) results showing variation in the species composition of the plots sampled from the piste and off‐piste. Each circle is a plot

Sixty vascular plants were recorded in the piste and 39 in the off‐piste. *Hypericum scabrum* and *Eremurus stenophyllus* were the dominant species in the piste and off‐piste areas, respectively (Figure 1). Ten dominant plants of each area are reported in Figure 2. Figure 3 shows the diagram of tb‐PCA. Moderate composition dissimilarity is observed between the piste and off‐piste.

### 3.2 Biodiversity

**Figure 4:**
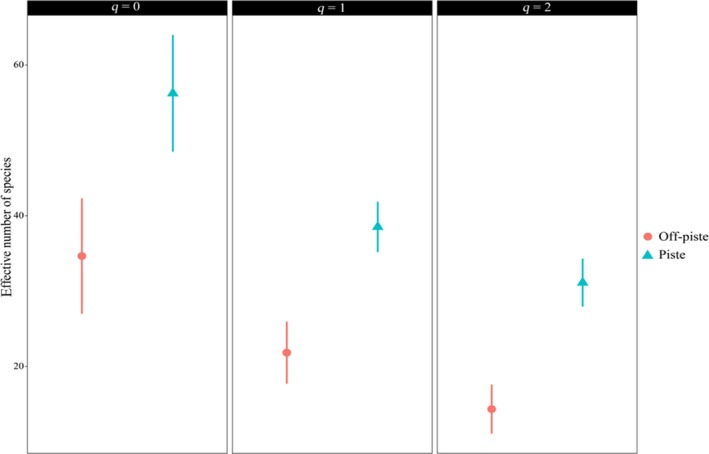
Hill species diversity of the two areas. The calculations were performed at the same sample completeness (i.e., 0.971). Species richness (*q* = 0), the exponential of the Shannon–Wiener index (*q* = 1), and the reciprocal of Gini–Simpson index (*q* = 2) are shown. These indices consider diversity at the level of rare, frequent, and dominant species, respectively. Error bars are the 95% confidence intervals of each index

The calculated coverage level for the two areas was 0.971. This value was used for the subsequent species and phylogenetic diversity calculations. The species richness (*q* = 0), the exponential of the Shannon–Wiener index (*q* = 1), and the reciprocal of the Gini–Simpson index (*q* = 2) of piste were significantly higher than that of the off‐piste (Figure 4).

**Figure 5:**
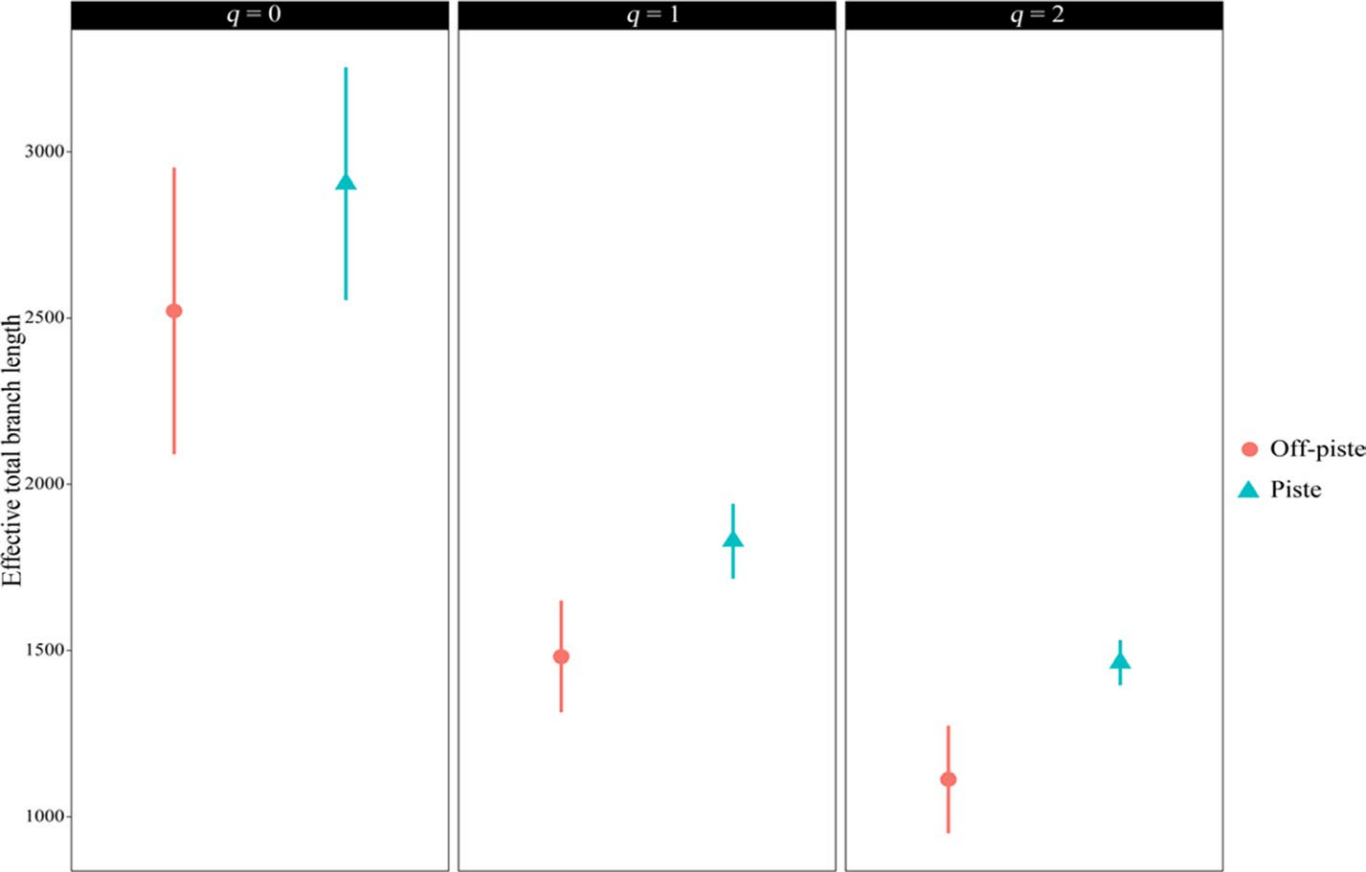
Phylogenetic Hill diversity of the piste and off‐piste areas. The calculations were performed at the same sample completeness (i.e., 0.971). Phylogenetic diversity at the level of rare (*q* = 0), frequent (*q* = 1), and dominant (*q* = 2) species are shown. The error bars are the 95% confidence intervals

Figure 5 shows that the phylogenetic Hill diversity of the two areas is similar in the zero order, and there is no significant difference between the piste and off‐piste as far as this index is concerned. However, the first and second order phylogenetic Hill diversity of the piste is higher than that of the off‐piste. Their confidence intervals are not overlapping, so there are significant differences among the piste and off‐piste considering these two indices.

**Figure 6:**
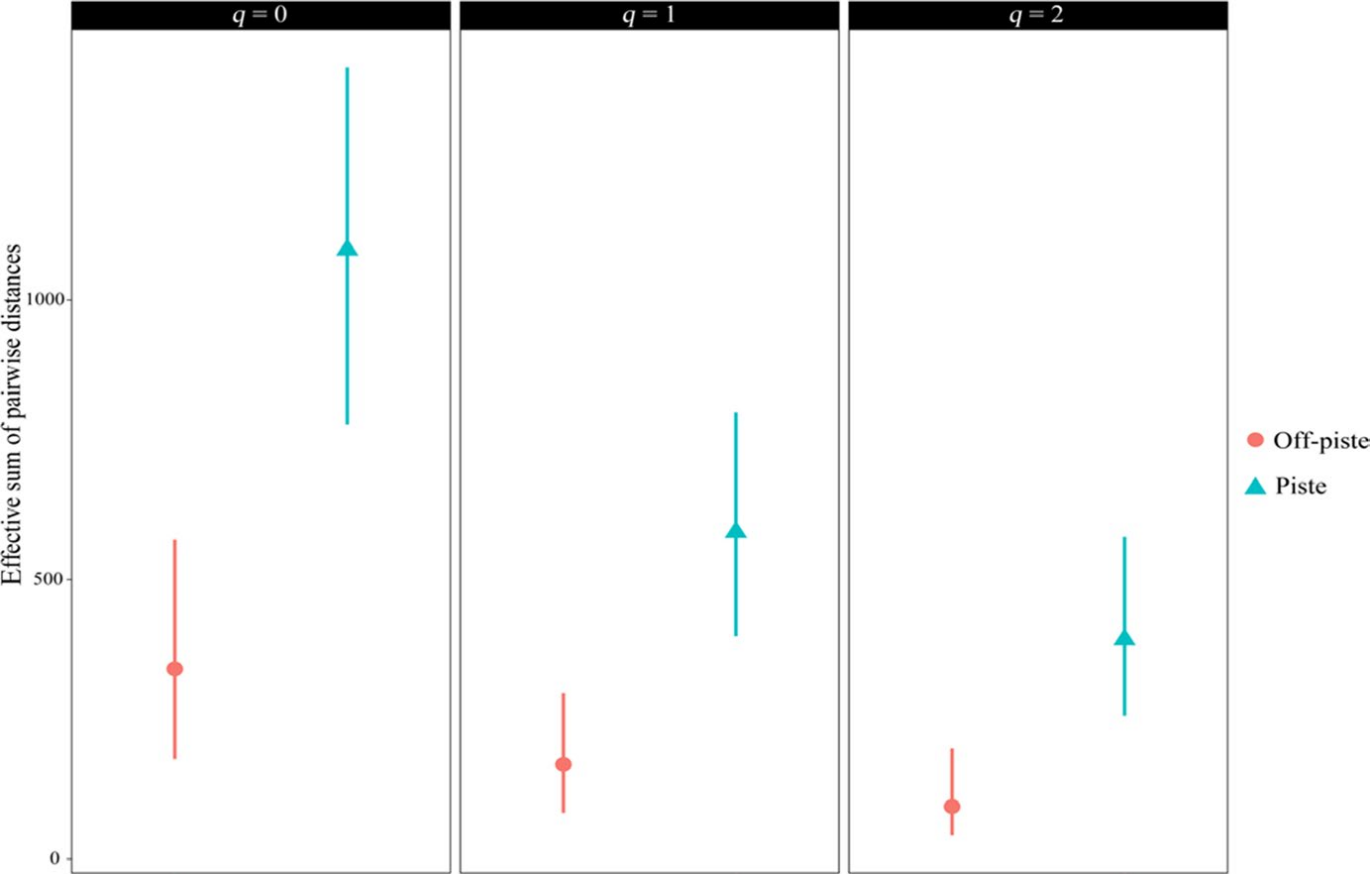
Functional Hill diversity of the piste and off‐piste. The calculations were performed at the base sample number. This result may be affected by sampling bias. Functional diversity at the level of rare (*q* = 0), frequent (*q* = 1), and dominant (*q* = 2) species are shown. The error bars are the 95% confidence intervals

The functional diversity of the piste was higher than that of the off‐piste at the three orders (i.e., *q* = 1, 2, and 3) of functional Hill diversity. It was indicating that functional diversity at the levels of rare, frequent, and dominant species are improved. These results are presented in Figure 6.

## 4 DISCUSSION

A piste and a degraded neighbor landscape were chosen for this research. Overgrazing was the primary disturbance event in both areas for an extended period. During the last 10 years, Shirbad piste and neighboring degraded landscape have experienced different management. Similar to the “nature‐friendly” management (Kašák et al., 2013), no artificial snow is used in the piste. However, small extent machine‐grading, late‐season grazing, and selective cutting of *Verbascum* sp. have been applied in the piste and were not reported by Kašák et al. (2013). We use EFPM term to distinguish between these management types. As stated, our goal is to examine whether EFPM could alleviate the effects of land degradation caused by long‐term overgrazing.

### 4.1 Effects of EFPM on the species composition

Cushion plants (i.e., *Acanthophyllum glandulosum* and *Acantholimon avenaceum*) along with *Cousinia* species are the indicators of a high‐elevation habitat type. Unpalatable species like *Eremurus stenophyllus*, *Hypericum scabrum*, *Artemisia deserti*, and *Euphorbia* species representing overgrazing history of the study area (Figure 2). Comparing the RIV graphs of the two areas, it is revealed a few dominant species and many rare species in the off‐piste. This type of abundance distribution is typical of disturbed habitats (Ejtehadi, Sepehry, & Akkafi, 2013). Relative homogenous distribution of RIVs in the piste suggests habitat heterogeneity with many frequent (not dominant) plants growing in the area.

*Euphorbia bungei*, *Bromus tectorum*, *A. deserti,* and *Cousinia freynii* were not recorded in the piste. Some of these plants are typically grown in lower elevations. Snow management in the piste might be the reason for this observation (Allegrezza et al., 2017). In our opinion, two important scenarios can be inferred from this observation; first, disturbances (e.g., overgrazing) can worsen the effects of climate change on the invasion of low‐elevation plants to alpine elevations (Lembrechts et al., 2017); second, in spite of the habitat degrading effects of pistes, these areas can be used for the alleviation of the climate change effects on the species distribution at the landscape scale.

Two palatable species, *Bupleurum falcatum* and *Melica persica*, along with *Euphorbia microsciadia* take the most advantages from EFPM. These dominant species in the piste were not recorded in the off‐piste. Moreover, some rare plants (i.e., *Elymus longearistatus* and *Astragalus sp*.) were recorded only in the piste. This also could be considered as an outcome of EFPM which allowed some seed bank species to grow in the area and shed lights on the dark diversity of the area (Pärtel, Szava‐Kovats, & Zobel, 2011).

Ten years of EFPM led to the differentiation of species composition in the area (Figure 3). Snow management and late‐season grazing could be seen as the drivers of this differentiation, however, further studies are required to verify this idea. The variation among the samples in the piste suggesting that EFPM led to habitat heterogeneity in the piste landscape.

### 4.2 Biodiversity changes after 10 years

Species richness (*q* = 0) is significantly higher for the piste in comparison with the off‐piste area. Land‐use change and light grazing might promote the species richness in that area. Mu, Zeng, Wu, Niklas, & Niu (2016) and Niu, Yang, Wang, Liu, & Hua (2018) also reported the species richness was higher in the light grazing, compared to the intensive grazing. Ten years of different management resulted in increased species diversity at the level of frequent (*q* = 1) and dominant species (*q* = 2; Figure 4). Our results are different from Wipf et al. (2005) and Roux‐Fouillet et al. (2011) that reported a significant species diversity decrease in the pistes. Different management of pistes is the key factor for this difference. Moreover, their off‐piste sites are relatively intact areas, but our off‐piste samples were taken from degraded land. Similar floristic diversity between the piste and off‐piste sites was reported by Allegrezza et al. (2017).

The zero order of phylogenetic Hill diversity of the two areas is similar (Figure 5). This finding may be due to the intensive grazing history, or the unfavorable climate conditions of the areas—the presence of strong environmental filtering in two landscapes leads to similar phylogenetic community composition (Shooner et al., 2018). In short‐term, EFPM howsoever could not lead to the improvement of plant phylogenetic richness. The significant differences in the first and second orders of phylogenetic Hill diversity of the landscapes are a good indicator of the improved phylogenetic diversity at the level of frequent and dominant native plants in the piste. In other words, EFPM has caused phylogenetically distinct taxa to dominate the area.

Functional richness indicates the extent of niche capacity that is inhabited by species in a community (Legras, Loiseau, & Gaertner, 2018; Mason, Mouillot, Lee, & Wilson, 2005). The functional diversity has been improved after the EFPM when it is compared to the off‐piste area (Figure 6). Our results suggest EF piste management has mitigated plant community disturbance in the area. Additionally, these findings suggest that late‐season grazing is a better choice for maintaining the ecosystem functions and services in the area.

### 4.3 Limitations

Finally, two potential limitations need to be considered. First, the unequal number of samples in the piste and off‐piste areas might affect functional diversity comparisons results. The coverage‐based approach that was used for species and phylogenetic diversity calculations completely solves this problem, but it is not introduced for the functional Hill diversity. Second, it was not possible to compare the biomass in the two areas due to the facts that we chose a nondestructive approach.

## 5 CONCLUSIONS

We have obtained comprehensive results proving that after 10 years of EFPM, plant communities in the piste have a higher diversity than those in the off‐piste. Differentiation in species composition is ongoing between the piste and off‐piste areas. Altering grazing time and controlling the number of grazers that enters in the degraded areas are the two managements that we strongly advise to be conducted in the area. The long‐term impacts of EFPM are still unknown. Future studies in the piste considering soil content change and fauna of the area are necessary before advising this type of piste management as an economic restoration practice.

## CONFLICT OF INTEREST

None declared.

## AUTHOR CONTRIBUTIONS

H.E., J.V., and M.B.E. had designed the early framework of the research. F.M. and H.M. modified the research framework and also identified the collected plants. M.B.E and M.F. collected the data. M.B.E. analyzed the data and wrote the first draft. The final manuscript is revised and approved by all of the authors.

## Data Availability

The data of this study are available on the Dryad with the https://doi.org/10.5061/dryad.4sb6383.
